# High Spatial Resolution Simulation of Sunshine Duration over the Complex Terrain of Ghana

**DOI:** 10.3390/s19071743

**Published:** 2019-04-11

**Authors:** Mustapha Adamu, Xinfa Qiu, Guoping Shi, Isaac Kwesi Nooni, Dandan Wang, Xiaochen Zhu, Daniel Fiifi T. Hagan, Kenny T.C. Lim Kam Sian

**Affiliations:** 1 College of Atmospheric Sciences, Nanjing University of Information Science and Technology, Nanjing 210044, China; mustapha.adamu@monash.edu (M.A.); kennylim@nuist.edu.cn (K.T.C.L.K.S.); 2 College of Applied Meteorology, Nanjing University of Information Science and Technology, Nanjing 210044, China; 18751971206@163.com (D.W.); xiaochen.zhu@nuist.edu.cn (X.Z.); 3 College of Geographical Sciences, Nanjing University of Information Science and Technology, Nanjing 210044, China; shiguopingnj@163.com (G.S.); nooni25593@alumni.itc.nl (I.K.N.); dans7messiah@nuist.edu.cn (D.F.T.H.)

**Keywords:** sunshine duration, sunshine percentage, complex terrain, remote-sensing, Digital Elevation Model (DEM), Ghana

## Abstract

In this paper, we propose a remote sensing model based on a 1 × 1 km spatial resolution to estimate the spatio-temporal distribution of sunshine percentage (SSP) and sunshine duration (SD), taking into account terrain features and atmospheric factors. To account for the influence of topography and atmospheric conditions in the model, a digital elevation model (DEM) and cloud products from the moderate-resolution imaging spectroradiometer (MODIS) for 2010 were incorporated into the model and subsequently validated against in situ observation data. The annual and monthly average daily total SSP and SD have been estimated based on the proposed model. The error analysis results indicate that the proposed modelled SD is in good agreement with ground-based observations. The model performance is evaluated against two classical interpolation techniques (kriging and inverse distance weighting (IDW)) based on the mean absolute error (MAE), the mean relative error (MRE) and the root-mean-square error (RMSE). The results reveal that the SD obtained from the proposed model performs better than those obtained from the two classical interpolators. This results indicate that the proposed model can reliably reflect the contribution of terrain and cloud cover in SD estimation in Ghana, and the model performance is expected to perform well in similar environmental conditions.

## 1. Introduction

Sunshine duration (SD) is an essential indicator that regulates the amount of solar radiation on the surface of the Earth. Recently SD studies have gained much attention because SD variability may affect energy balances (through rates of photosynthesis) and temperature, as well as regulating the rate of evapotranspiration, and therefore posing a significant threat to the water, energy and carbon cycles of the Earth’s system [1,2,3]. As an important meteorological parameter, SD is known to correlate well with surface-radiation budget and is useful in many applications [1,2,4,5]; however, in-depth knowledge of changes in SD as a good predictor (of solar radiation) have not been fully explored by the Global Climate Observing System [6,7]. This is because SD is highly variable in space and time, and its trend is not fully explored, particularly in regions where data collection is limited in spatial and temporal coverage [8]. The accurate information of SD (a predictor of solar radiation intensity) under different natural landscape scenarios is of value to solar energy-based projects, cost-benefit analyses and the evaluation of long-term performances of solar energy conversion systems [9,10]. Also, accurate information about SD and the role that it plays in surface-radiation variability would represent a valuable contribution to existing knowledge, and could improve our understanding of the global surface energy budget.

Ghana, an emerging economy in West Africa, is experiencing energy generation deficits (caused by low water levels to run hydro-electric dams and an increased cost of fossil fuels). This generation shortfall has resulted in an acute power crisis over the period 2000–2017, and has subsequently downgraded Ghana’s investor confidence and credit ratings [11,12]. On the other hand, due to its geographic location, Ghana enjoys bright and high levels of sunshine throughout the year, and the opportunity to effectively exploit and develop renewable energy technologies such as solar energy to promote the ongoing campaign of a clean environment is promising (as energy is intrinsically linked with environmental, social and economic dimensions of sustainable development). The country’s annual average global horizontal radiation varies from 2.0 to 9.0 kWh/$m^{2}$/day, and the sun shines for about 365 days in a year. The national average sunshine hours and solar energy are 5–5.7 h/day and 4.4–5.6 kWh/$m^{2}$/day, respectively [13].

Additionally, economic and energy-policy experts have suggested that the solar radiation needed to convert sunlight into energy is sufficient to reduce Ghana’s energy-generation deficits substantially [11,12]. Furthermore, the photovoltaic devices have cost-saving benefits, when utilized in the telecommunication industry, for pumping rural water supply systems, rural electrification projects, and so on. Given these numerous possible applications and benefits of solar energy-utilization technologies to Ghana’s energy sufficiency, Ghana’s investment in solar energy systems is still very low (i.e., 2.5 MW of solar PV plant or 0.1% of total electricity generation) and unable to efficiently utilize the power of the Sun as an alternative energy source. One reliable reason is that information about the direct measurements of solar radiation distribution throughout the country is limited to very few synoptic stations. To design solar-based projects or evaluate solar-system performance, we need long-term and reliable monthly and daily average solar radiation data, which are not presently available from the Ghana Meteorological Agency (GMA) due to financial and technical constraints (i.e., the high cost and maintenance requirement of installing additional equipment) [14,15].

To estimate solar radiation distribution in Ghana throughout the year based on other meteorological parameters, scientists have developed models such as empirically based models, remote-sensing retrievals [16,17,18,19,20], artificial intelligence [17,21,22,23] and single-layer and multi-layer radiative transfer models [24]. Traditionally, empirically based models have been mostly preferred due to their accessibility and cost of computation [25], and have been commonly utilized to predict solar radiation in agriculture [16], water resources management [15] and solar energy systems [26]. The choice and selection of a specific type of empirical model depends on the accuracy required, and on the available meteorological parameters. Empirical models such as sunshine-based [27,28,29], temperature-based [30,31] and cloud-based models [14] have been proposed. This study does not attempt to provide a description of the many classes of the available solar radiation models, as that has already been provided in other papers [32,33,34].

Global solar radiation has been measured at some parts in Ghana [9,10]. For example, Arku [9] modelled monthly daily average global solar radiation in four cities, namely, Akuse, Bole, Kumasi and Wenchi. Quansah et al. [10] used SD and temperature-based models to predict monthly average daily global solar radiation for Owabi in the Ashanti region of Ghana. These studies tested their methods (i) over horizontal terrains, (ii) over a few or small regions of Ghana and (iii) over the entire country of Ghana based on classical interpolators and without taking into account the complex terrain and atmospheric factors. Meanwhile, there have been some models that utilize satellite data (e.g., geostationary satellite observations, the digital elevation model (DEM)) and meteorological parameters (e.g., SD) to improve solar radiation estimations.

SD measurements at any location are influenced by terrain factors (e.g., slope, aspect and terrain shadowing degree) and atmospheric factors (e.g., air molecules, aerosols, moisture, cloud cover and cloud types). For example, Zhu et al. [35] developed a remote sensing model that incorporated the digital elevation model (DEM) to reflect these features and incorporate sunshine percentage (estimated by the moderate-resolution imaging spectroradiometer (MODIS)) and cloud products (to broadly reflect atmosphere), to compute SD in the Ningxia Hui Autonomous Region. Pons and Ninyerola [36] estimated solar radiation based on a remote-sensing model in Catalonia, Spain, and found that including effects of topography and meteorological parameters improved the solar radiation estimates over complex terrain. Matzarakis and Kasoulis [37] also estimated SD for Greece and the results showed that the seasonal variation in SD estimates were influenced by topography. However, based on our best knowledge and the previous relevant studies on Ghana, the effect of complex terrain and atmospheric components have not been considered for estimating SD and subsequently modeling solar radiation. Thus, based on the methodology applied in previous studies, this study incorporates the effects of both complex terrain and atmospheric factors by using the DEM data for Ghana and daily MODIS cloud products for 2010 to construct a remote-sensing model to investigate the temporal and spatial distribution of SD over Ghana. The study further evaluates the proposed model performance against two widely used classical interpolators (i.e., inverse distance weighting (IDW) and kriging). Although this study is an improvement over previous works, this proposed methodology has rarely been applied in the West African sub-region, and its application in Ghana is of great importance because (1) it covers more recording sites, and (2) a reliable and sophisticated geo-statistical model over complex terrain is being utilized. The use of remote-sensing based measurements to develop an SD model over the complex terrain of Ghana may provide an additional understanding of the dynamics of SD, which strongly correlate with solar radiation. 

Section 2 gives a description of the data and methods used in this study. An evaluation of the annual and seasonal satellite-based SD estimates compared against ground-based data is shown in Section 3. The results and discussion of the proposed model and its comparison with other classical interpolators are discussed in Section 3. The conclusions and recommendations are presented in Section 4.

## 2. Materials and Methods

### 2.1. Data

The study used three kinds of datasets, as follows.

#### 2.1.1. In Situ Observations

The study acquired daily observed sunshine hours from GMA. Sunshine hours data was used as a proxy to compute SD for the year 2010. The daily sunshine hours data was averaged into annual and monthly values and converted to SD for model development and performance assessment. Overall, data from 22 sites were collected and used (Table 1).

#### 2.1.2. Remote Sensing and Other Supplementary Data

The study acquired daily TERRA/MODIS (MOD06−L2) and Aqua/MODIS (MYD06−L2) cloud data for 2010, with a spatial resolution of 5 × 5 km. The dataset was downloaded from the NASA webpage at https://search.earthdata.nasa.gov/ (accessed: 10 October 2017). To obtain the total daily cloud cover, the following pre-processing, geometric correction, image mosaic and total daily clover image superposition procedure were performed. The purpose of geometrically correcting the satellite data, image mosaic and clover image superposition are extensively discussed in literature [38,39]. The national boundary shapefile (Vector data; Diva-GIS shape file) was acquired from Diva-GIS website at Diva-GIS [29] (http://www.diva-gis.org/gdata).

A Shuttle Radar Topography Mission (SRTM) Digital Elevation dataset with 1 x 1 km resolution was acquired from NASA LP DAAC (http://dx.doi.org/10.5067/MEaSUREs/SRTM/SRTMGL1.003) due to high quality performance [40,41]. The data were downloaded for the whole of Ghana (accessed: 4 November 2017). The mosaic function in ArcMap GIS was used to merge the multiple tiles. The DEM data and the MODIS cloud data for 2010 were respectively extracted by mask (a GIS function) using the Ghana boundary shapefile in ArcMap. To incorporate both the DEM and MODIS data in the model, the 5 × 5 km MODIS dataset was resampled to 1 × 1 km to match that of the DEM data by using nearest neighbor resampling method. The nearest neighbor resampling method assigns the digital number (DN) value of the closest original pixel to the new pixel while preserving and retaining all spectral information, making the method the most popular and preferred method in many studies [42]. 

All datasets were processed using the following software: ArcGIS (the Environmental Systems Research Institute, Inc.’s geographic information software), ENVI (Environment for Visualizing Images) and Matlab. ArcGIS was used for the projection and its secondary development, ENVI was used for remote-sensing image analysis and display and Matlab was used for the statistical analysis of the data. The study applied strict quality checks and controls to all datasets used.

### 2.2. Methods

#### 2.2.1. Sunshine Percentage (SSP) Estimation Model

Based upon this linear relationship between SSP and cloud fraction [43], a linear regression model was used to calculate the sunshine percentage from the regression equation below.

(1) SSP=a+bc

where SSP is the sunshine percentage with a monthly temporal resolution for the year 2010, *a* and *b* are regression constants and *c* represents the mean monthly cloud cover obtained from MODIS AQUA and TERRA platforms (MOD06_L2 and MYD06_L2).

#### 2.2.2. The SD Model

The SD was computed by multiplying the maximum possible sunshine duration (MPSD) by the SSP, therefore resulting in the monthly sunshine duration over complex terrain for the year 2010. The model equation is stipulated below.

The SD for complex terrain was calculated as follows:

Lαβ = Lgαβ × SSP,(2)

where, Lαβ is the SD of complex terrain, and Lgαβ is the maximum possible sunshine duration (MPSD) of complex terrains. Lgαβ was calculated using the MPSD distribution model developed by [44] for complex terrains, and SSP was derived from the sunshine percentage estimation model described above. 

#### 2.2.3. Spatial Interpolation Methods

Two commonly used spatial interpolation methods were used in this study, namely, inverse distance weighting (IDW) and kriging.

A. Inverse distance weighting (IDW)

Inverse distance weighting (IDW) is a straightforward and less computationally intensive interpolation method. The IDW interpolation used in this study was implemented in ArcGIS version 10.3 software. Using the ArcGIS Geostatistical Analyst tool, the study followed the standard spatial interpolation procedures explained in Lu and Wong [45]. The IDW method assumes that an observation from a sampled point $\left(Z_{i}\right)$ is closer to other un-sampled points $\left(Z_{j}\right)$ than values from samples farther apart, as expressed mathematically in Equation (3). To predict values from un-sampled locations, the study used monthly SD observation values from 10 out of the 22 synoptic stations of Ghana for 2010.

(3) $$Z_{j}=\frac{\sum_{i=1}^{n}w_{ij}Z_{i}}{\sum_{i=1}^{n}w_{ij}}$$

where n represents the number of sampled points, $Z_{i}$ is the measured values at the sampled point i, $Z_{j}$ is the estimated value at grid location j and $w_{ij}$ is the weight that controls the effect of the control points on the estimation of $Z_{j}$. $w_{ij}$ is set equal to Euclidean distance ($d_{ij}^{-\alpha }$) between the referred sample point and interpolation point. The study used a value of 2 as the optimal power function (α) based on the recommendation of Armstrong and Marciano [46].

B. Kriging

In addition, the study used kriging to interpolate monthly SD values from 10 out of the 22 synoptic stations of Ghana for the 2010 period according to Equation (4), for the purposes of comparison. The methodology for Kriging was implemented in ArcGIS version 10.3 software following the standard spatial interpolation procedures by Mulholland et al. [47] and Sen and Sahin [48].

(4) $$f\left(x,y\right)=\sum_{i=1}^{n}\psi_{i}W_{i}$$

In both methods, each grid point (which represents a mean of over 1 km^2^ area), the monthly sums of the interpolated values were determined for every month of the corresponding series. The following months were considered: January, April, July and October. Finally, the regional mean values were determined by averaging the values obtained for all grid points for each month of the series.

#### 2.2.4. Evaluating Model Results

To assess and compare the performance of the model, the study used popular statistical metrics such as the mean absolute error (MAE), the mean relative error (MRE) and the root-mean-square error (RMSE) to test the linear relationship between estimated or simulated SD and ground-based SD estimates. The MAE is the average difference between simulated SD and ground-based estimates. The MRE indicates the mean error between simulated SD and ground-based estimates. The RMSE is a general indicator of the standard error between the simulated SD and ground-based estimates. The smaller the RMSE value, the better the model’s performance. The equations for estimating the statistical indicators mentioned above are as follows:

(5) $$MRE=n^{-1}\left(\sum_{i=1}^{n}\left|x_{i}-y_{i}\right|\right)\times 100\%$$

(6) $$MAE=n^{-1}\sum_{i=1}^{n}\left|x_{i}-y_{i}\right|$$

(7) $$RMSE=\sqrt{n^{-1}\sum_{i=1}^{n}{\left(x_{i}-y_{i}\right)}^{2}}$$

where *x_i_* is the simulated SD, *y_i_* is the in situ SD and *n* is the number of observations.

## 3. Results and Discussion

### 3.1. Variations of SSP

**Figure 1:**
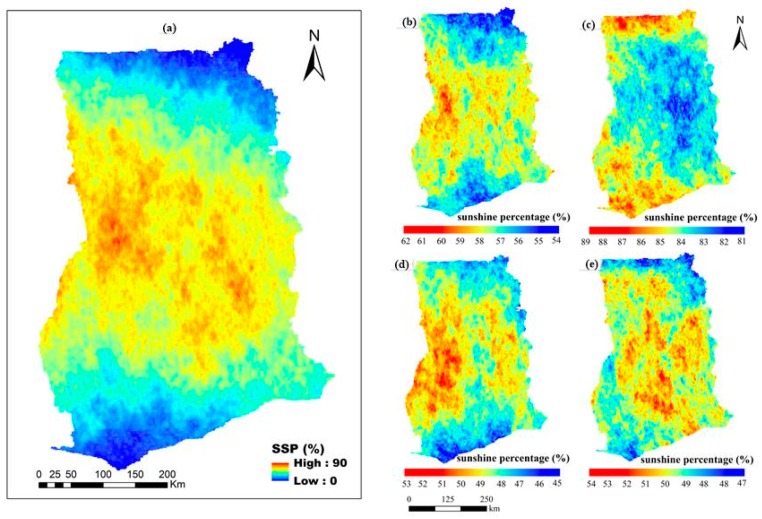
Sunshine Percentage (SSP) for (**a**) whole year, (**b**) January, (**c**) April, (**d**) July and (**e**) October, 2010. High SSP values indicate months with more days of sunshine and low SSP values indicate months with fewer sunny days.

The sunshine percentage estimates derived using remote sensing are analyzed for four selected months in the year 2010 and presented in Figure 1. Generally, the MODIS cloud fraction, which was used in calculating the SSP results, showed that a high amount of cloud cover leads to lower amounts of sunshine received at the Earth’s surface. Figure 1a shows the total SSP, and Figure 1b–e shows the SPP for January, April, July and October, respectively. For calculative purposes, spring, summer, autumn and winter were represented by April, July, October and January, respectively. From the figure, high SSP values indicate seasons (months) with more days of sunshine, and low SSP values indicates seasons (months) with less sunny days. The magnitude of monthly SSP is ranked as follows: April > January > July > October. The overall SSP results clearly indicate that winter (January) and spring (April) are characterized by higher SSP values than summer (July) and autumn (October). The high SSP in tropical environments during the dry season was expected, and is consistent with previous studies [18,19,43,45]. SSP values in April are higher than in January primarily because April is a transition month with decreased cloud cover during day, and increased cover during the night. However, the increased night cloudiness restricts or traps the outgoing long-wavelengths (OLRs) in contrast to January, part of the Harmattan season (a cloud-free season) that lowers humidity and creates hot days and cold nights. Low SSP values in July and October are attributed to increased cloudiness and rainy days. The magnitude of SSP is higher in northern Ghana than the southern part. Table 2 shows the results of the error analysis for simulated SD over the complex terrain of Ghana. The results indicate that April presented a maximum monthly MAE of 9.86% higher than the annual average (6.45%) in 2010.

### 3.2. Estimation and Variation SD

**Figure 2:**
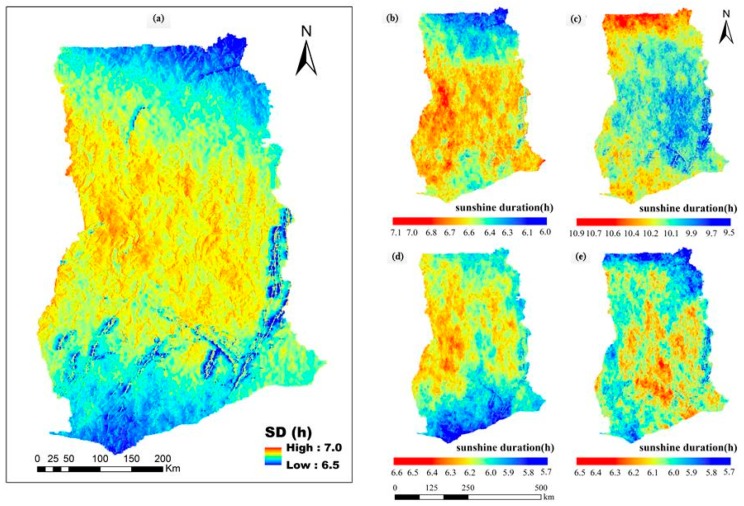
2010 SD for (**a**) annual, (**b**) January, (**c**) April, (**d**) July and (**e**) October, 2010. High values indicate long SD, and low values indicate low SD (in hours), respectively. SD lies between July (minimum) and April (maximum).

Figure 2 shows the spatial distribution of seasonal SD over the complex terrain of Ghana during the four seasons of 2010. As shown in Figure 2a, the annual average daily total of SD was between 6.5 and 7.0 h in 2010 in Ghana. The spatial distribution of the annual average daily total SD was at its maximum in the mountainous areas and plains in a wide strip across central Ghana, rather than in the north and south of the country. The maximum annual average daily total SD (7.0 h) was observed in the western mountainous and mid-eastern plains. The minimum annual average daily total SD (<6.7 h) was observed in northern and southwestern Ghana. 

SD also presented strong seasonal variations (Figure 2b–e). Generally, the relative magnitude of monthly average daily total SD for the various months was consistent with the SSP distribution over the study area, suggesting that regions with the highest monthly average daily total SSP corresponded to highest monthly average daily total SD, and vice versa. The seasonal variations are predominantly explained by the Sun’s position (i.e., Sun’s movement with respect to time) and shading effects provided by slope, aspect and surrounding terrain. April recorded a maximum monthly average daily total SD (10.66 h) specifically in the extreme north, with slightly higher values in southwestern areas than in low-lying areas of the southeastern part and middle belt of the country. In January, the extreme north and coastal belt received a minimum monthly average daily total SD (<6.1 h) whilst the maximum monthly average daily total SD was observed in the west and east. In July, the eastern plains and western mountain ranges received increased SD. The maximum SD was recorded in the western mountains followed by eastern plains. The coastal plains and extreme north received minimum SD values of 5.9–5.7 h and 6.2–5.9 h, respectively. In October, the highest SD was recorded in the enclosed plains in the central and eastern parts (>6.5 h). The SD in the southeast coastal plains increased, whilst the minimum average daily total SD was found in the extreme north (5.9–5.7 h) and southwestern coastal plains (6.1–5.9 h). 

To evaluate the simulated SD, the study evaluated the performance of the estimated mean monthly SD against ground-based data (for 2010) using performance statistics. Table 2 presents the results between ground-based estimates from the stations and the simulated SD: The results show that January, July and October 2010 show lowest MAE (0.36) while April had the highest MAE (0.91). On the other hand, results for MRE show that January (9.46%) performed better and April (20.47%) performed worse. One of the possible reasons could be that the month of April serves as a transition period and the dynamics might not be well captured in the proposed model, a hypothesis that requires future investigation.

### 3.3. Comparison of Simulated SD, IDW and Kriging Results

A summary of the model evaluations is provided in Table 3 for the simulated SD, IDW and kriging models, respectively. The results reveal that in all model evaluation methods, the proposed model, compared to the two classical interpolators, presented the smallest MAE, MRE and RMSE. The simulated SD appeared to perform better than both IDW and kriging. This finding is consistent with previous studies on China [35], Greece [37] and Spain [36], where topographic and atmospheric effects were considered. Future studies are needed to perform a comparative analysis of method to estimate solar radiation for the region.

**Figure 3:**
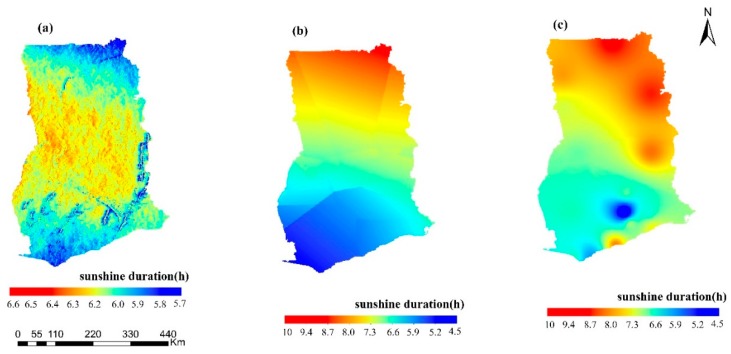
Comparison of SD obtained for 2010 (**a**) over complex terrain and using (**b**) IDW and (**c**) kriging.

The spatial distribution of sunshine duration for 2010 based on the models is presented in Figure 3. The spatial distribution of annual average daily total SD from the proposed model is different when compared to that from the IDW and kriging models. The proposed model shows maximum SD in the west and east rather than in the north and south. This is predominantly due to the effect of slope, aspect and surrounding terrain. IDW and kriging present an increasing annual average daily total SD from south to north. The longest annual average daily total SD for the IDW and kriging was 9.71 and 10.8 h, respectively, in northern Ghana. Kriging showed a higher SD in the north and northeast, and a slightly higher SD along the eastern coast of Ghana. 

### 3.4. Influence of Terrain on SD

**Figure 4:**
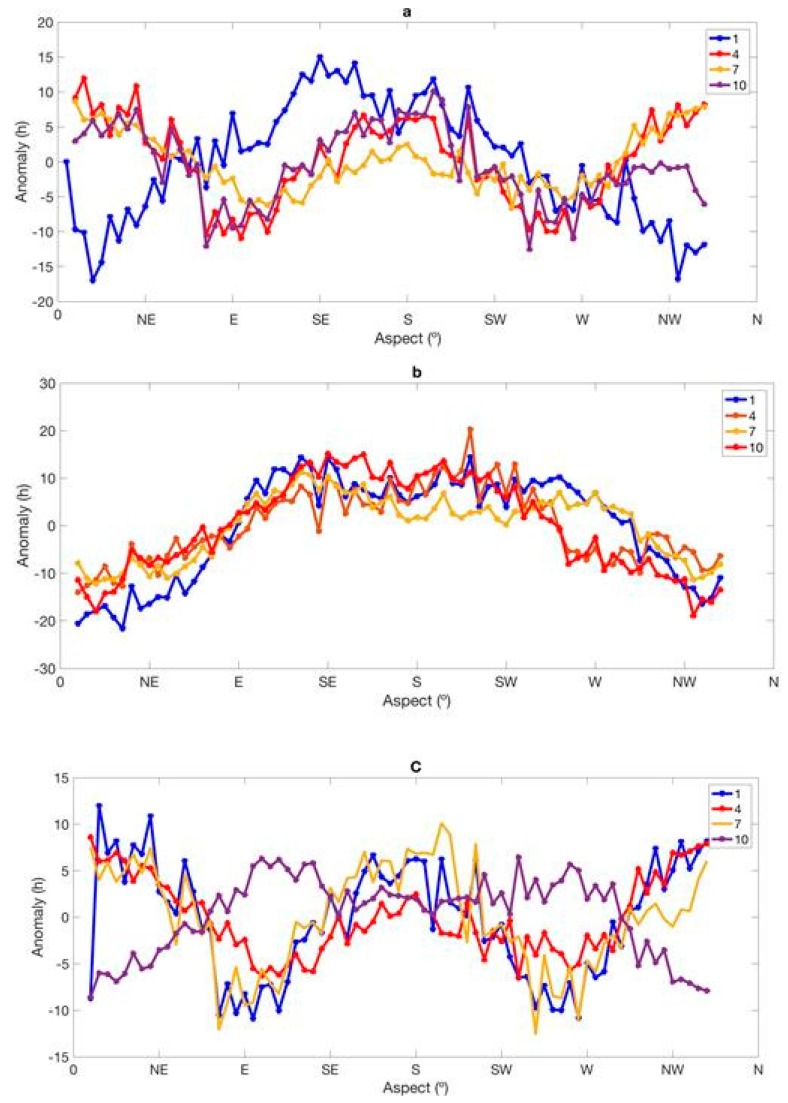
Anomaly curves with respect to slope direction of (**a**) maximum possible sunshine duration (MSPD), (**b**) SD and (**c**) SSP, for 1 January, 4 April, 7 July and 10 October 2010.

This study computed anomalies for selected grids located in mountainous areas to further elucidate the effect of topography at localized scales. The anomalies used here are defined as the difference between the slope direction of the selected grid box and the average slope direction for all the grid boxes. The study computed anomalies for selected grids located in mountainous areas to further explain the effect of topography at localized scales. This analysis was done for a latitude range of 4.5°–6.0° N and a slope between 5° and 9°. Moving in a clockwise direction, the spatial aspect of 90° denotes east, 180° denotes south and 270° denotes west [43]. Figure 4 represents the anomaly analyses of (a) MPSD, (b) SD and (c) SSP with respect to slope direction for the months of January, April, July and October. It can be seen that both maximum possible sunshine duration (MSPD) and SSP (Figure 4a,c) show different variations in anomaly. However, SD shows similar trends for all months (Figure 4b), which reflects the added value of DEM inclusion in our model. To simulate SD accurately, factors that affect the amount of SD that reaches the surface of the Earth, such as topography of the underlying surface, obstruction of SD from adjacent elevations and the solar angle at a given time, must be inculcated in the model simulation. By using a complex computational algorithm (see [44] for more details), our model was able to inculcate all these factors in SD simulation, thereby making it robust when compared to both IDW and kriging (see [49]). For the southern slope direction, January had the highest slope, followed by October, then April and finally July. This confirms that more sunshine was received during the January and April months than the July and October months. They generally had an ascending trend from 0–90°, then ascension continued from 90–180° and descended from 270–360°.

## 4. Conclusions

A remote-sensing model to estimate the spatio-temporal distribution of SSP and SD was built for Ghana, taking into account topography and MODIS cloud products in 2010. The annual and monthly average daily total SSP and SD were estimated based on the proposed model. The model-estimated SD was evaluated against in situ observation datasets based on error analyses of mean absolute error (MAE) and mean relative error (MRE). The error analyses indicated that the simulated SD was in good agreement with ground-based estimates. Also, the study evaluated the performance of the proposed model with two classical interpolators (kriging and IDW). The performances of the models were determined using MAE, MRE and RMSE. Overall, the relative magnitude of annual and monthly variations in SSP were consistent with those from previous studies within the country. The highest monthly SSPs were observed in April and January due to longer sunshine hours. The lowest monthly SSPs in July and October were largely attributed to cloudier and rainier days. The spatial pattern of the annual SSP and annual average daily total SDs were higher in the western mountainous and eastern plain areas than in the north or south. However, the seasonal distribution of the annual SSP and annual average daily total SD were found to be higher in the western mountainous areas and in the north than in the east or south. The results also revealed that the SD obtained from the proposed model performed better than the SD obtained from classical interpolators (i.e., with the smallest values of MAE, MRE and RMSE of 1.07, 0.19 and 1.18, respectively). The results show that the remote-sensing model can predict the pattern of SD for Ghana very well. Therefore, a new remote-sensing model has been developed to estimate SD for Ghana, or regions with similar environmental conditions where direct solar radiation data are limited or unavailable. The results from this study will contribute to advancing SD studies and, consequently, to improving estimation of solar radiation for solar energy assessment at a national level.

## Figures and Tables

**Table 1:** ID, station name, location, and elevation of 22 available synoptic GMA stations in Ghana.

World Meteorological Organisation (WMO) ID (Prefix-654)	Station Name	Latitude	Longitude	Altitude (m)	Region
50	Abetifi	06°40′N	00°45′W	594.7	Eastern
72	Accra	05°36′N	00°10′W	67.7	Greater
75	Ada	05°47′N	00°38′W	5.2	Greater
62	Akatsi	06°07′N	00°48′W	53.6	Volta
57	Akim Oda	05°56′N	00°59′W	139.4	Eastern
60	Akuse	06°06′N	00°07′W	17.4	Eastern
65	Axim	04°52′N	02°14′W	37.8	Western
16	Bole	09°02′N	02°29′W	299.5	Northern
53	Ho	06°36′N	00°28′W	157.6	Volta
37	K’ Krachi	07°49′N	00°02′W	122.0	Volta
59	Koforidua	06°05′N	00°15′W	166.5	Eastern
42	Kumasi	06°43′N	01°36′W	286.3	Ashanti
01	Navrongo	10°54′N	01°06′W	213.4	Upper East
69	Saltpond	05°12′N	01°04′W	43.9	Central
45	S’ Bekwai	06°12′N	02°20′W	170.8	Western
39	Sunyani	07°20′N	02°20′W	308.8	Brong Ahafo
67	Takoradi	04°53′N	01°46′W	4.6	Western
18	Tamale	09°33′N	00°51′W	168.8	Northern
73	Tema	05°37′N	00°00′W	14.0	Greater
04	Wa	10°03′N	02°30′W	322.7	Upper West
32	Wenchi	07°45′N	02°06′W	338.9	Brong Ahafo
20	Yendi	09°27′N	00°01′W	195.2	Northern

**Table 2:** Summary of error analysis for simulated sunshine duration (SD) over the complex terrain of Ghana.

Month	1	4	7	10	Average
Mean absolute error (MAE) (h)	0.36	0.91	0.72	0.48	0.61
Mean relative error (MRE) (%)	9.46	20.47	14.66	14.08	13.14

**Table 3:** Summary of model evaluation using Inverse distance weighting (IDW) and kriging.

Attributes	Root-Mean-Square Error (RMSE) (h)	MAE (h)	MRE (%)
Proposed	1.18	1.07	0.19
IDW	1.78	1.72	0.28
Kriging	2.25	2.21	0.37
